# Examination of the Brain-Dead Organ Donor Management Process at a Spanish Hospital

**DOI:** 10.3390/ijerph15102173

**Published:** 2018-10-04

**Authors:** Antonio Sánchez-Vallejo, Juan Gómez-Salgado, María Nélida Fernández-Martínez, Daniel Fernández-García

**Affiliations:** 1 Transplant Coordinator, University Health Complex of León (CAULE), SACYL, 24071 León, Spain; asanv@unileon.es; 2 Nursing Department, University of Huelva, 21071 Huelva, Spain; 3 Safety and Health Posgrade Program, Universidad Espíritu Santo, Samborondón (Guayaquil) 091650, Ecuador; 4 Health Sciences School, Biomedical Sciences Department, Pharmacology Area, University of León, 24071 León, Spain; nelida.fernandez@unileon.es; 5 Health Sciences School, Nursing and Physiotherapy Department, University of León, 24071 León, Spain; dferg@unileon.es

**Keywords:** transplant coordination, detection, organ donor, brain death, emergency services

## Abstract

The number of donors and organs available has not increased at the same rate as the inclusion of new patients in the waiting lists. The aim of the present study was to analyze the quality of the detection process of potential brain-dead organ donors at the Hospital of León. For this, a cross-sectional prospective study was developed on a retrospective cohort of patients who were admitted or died by catastrophic brain damage with the potential for organs donation. Data were collected for six months using hospital records of admissions and exitus. For the statistical analysis, the free distribution software Epi Info 3.5.4 was employed. A total of 627 patients were studied: 550 were discharged and 77 died as potential donors. Of the potential donors, 65 died in asystole, but 60 of them had an absolute contraindication to donation and 20 died after limitation of life support therapy. Five cases with donor criteria in controlled asystole were detected. The analysis found that the detection process conformed to the regulatory framework stablished by the National Transplant Organization. However, population aging leads to a high rate of absolute contraindications among detected potential donors. The donation capacity of the hospital could therefore be increased with the implementation of a donor protocol in controlled asystole.

## 1. Introduction

Organ transplantation is a therapy that restores the health of patients and enables them to return to their daily activities [1]. Organ donation substantially increases the life expectancy of patients; some authors claim that the percentage of potential years of life gained for a set of receptors of a six multiorgan donation is 55.8%. It has also been found that organ transplantation is a highly efficient procedure from a cost-effective point of view, which is also positive for the health system [2].

The social and economic benefit associated with the continuous improvement in postimplant survival results has led to an increase in transplant indication as a therapeutic alternative of first choice for a large number of pathologies [1,3,4,5]. However, the number of donors and the organs available has not increased at the same rate as the inclusion of new patients in the waiting lists [3,4,6].

This effect has led to a lengthening of access time for transplants, resulting in a negative impact on the quality of life and chances of survival for these patients; it is estimated that between 6% and 8% of patients die on the waiting list [7,8,9].

With rates of donation close to 40 donors per million population (pmp) (39.7 in 2015) [10], the Spanish donation and transplant model has become the most favorable worldwide and is being successfully exported to various countries [3,4,7]. A decisive factor for this success has been the development of a regulatory framework, which guaranteed the highest levels of equity, quality, and safety in the process [11,12,13,14]. For this, the National Transplant Organization (Organización Nacional de Trasplantes: ONT for its acronym in Spanish, henceforth referred to as such) has kept—since 1998—a rigorous quality assurance program in the process of donation (Programa de Garantía de Calidad en el Proceso de Donación: PGCD for its acronym in Spanish, henceforth referred to as such), which has greatly contributed to the continued rise in the donation rate [15].

Based on the PDSA (Plan-Do-Study-Act) methodology of learning and improvement, which has irrefutable benefits when applied to healthcare environments [16], the PGCD allows for the definition of the theoretical organ donation ability of each hospital, the areas of improvement in potential donors detection, hospital factors with the greatest impact on the process of donation, and the provision of improvement and evaluation options [15,17]. The cornerstones of the PGCD for nontransplant hospitals, such as the University Health Complex of León (CAULE), include systematic monitoring of all potential donors that appear in each hospital, a decrease in family refusals, and a minimization in the loss of donors during detection, assessment, and maintenance [4,8,11,17,18,19].

The figure of the Hospital Transplant Coordinator (HTC) is a key element in the entire donation process and quality control [3,20]. The main purpose of the HTC is to obtain organs for transplants and the continuous improvement of donation rates, but this figure also has the ultimate responsibility for all aspects relating to the donation and transplant process, in particular the detection of potential donors [3]. The detection phase, due to its complexity and significance, is the initial and the most important step of the process, and it is the area that requires greatest emphasis on quality control in nontransplant hospitals [20].

Based on this evidence, the objective of this study was to analyze the quality of CAULE brain-dead potential organ donor detection process using a study designed and validated by the hospital itself.

## 2. Materials and Methods

### 2.1. Context

Donation after brain death (BD) has significant potential as a source of donors (from seven to eight) despite the increase of donation in asystole [21]. In 2015, more than 80% of donors in Spain died by brain death (BD), with a profile of males with a mean of 60 years old who died after a stroke. The average is 69 years in the Castilla y León autonomous region, where CAULE is located [10,22,23].

This hospital is a type-two centre according to the classification of accredited hospitals for donation and transplant [20,22]. By 2015, it was endowed with 1051 beds, obtained a donation rate of 50.6 donors pmp, and gave coverage to a population of 335,770 inhabitants [22]. It has four Critical Care Units (CCU), adding up to 44 beds: 16 beds in the adults multipurpose Intensive Care Unit (ICU), four in the Pediatric ICU (PICU), 12 in Critical Postsurgical Resuscitation (RES), and 12 in the Critical Coronary Care Unit (COR). It also has extra-CU units with the potential of generating donors, according to the consulted literature: Neurology Service Stroke Unit (NRL), Neurosurgery Service (NRS), Internal Medicine Service (IM), and Emergency Service (EME) [19,24].

### 2.2. Design

The study was a cross-sectional prospective descriptive study of a retrospective cohort oriented to the improvement of detection (identification and notification to the HTC) of catastrophic brain damage patients with the potential to be organ donors at their admission process or during their hospital stay.

### 2.3. Relevant Definitions

Catastrophic brain damage (CBD): severe brain structural and functional damage of a traumatic, stroke, tumor, infectious, or otherwise origin, accompanied by a coma defined by a Glasgow Coma Score (GCS) lower than 8 [24,25].

Potential donor (PD): CBD patient who has died in a clinical situation compatible with brain death (BD), asystole, or after a limitation of life support therapy (LLST), with the potential to become an organ donor [21,26,27].

### 2.4. Sample

The sample selection followed a nonprobability discretionary pattern. For six months (1 January to 30 June 2016), a daily scrutiny of all avenues reported to CAULE was conducted through the Emergency Service and the admissions in the Critical Care Units not coming from the Emergency Service. The number of exitus were registered every day, establishing contact with the potential donor generating units when any clarification on some record was needed.

Patients who met the inclusion criteria, similar to that found in analogous studies and in the quality assurance program in the process of donation of the National Transplant Organization [8,15,16,17,18,20,26,28] were selected:

- Patients with presence of injury that was compatible with CBD at admission or appearance of the same during hospitalization or the performance of any medical procedure (confirmed by neuroimaging test or discharge or death report).
- Exitus recorded in any hospital unit (including EME and COR) where the primary or secondary diagnosis of death was compatible with the existence of CBD.

### 2.5. Variables

Dependent or result variables were donation after BD at the end of the screening process, understood as the definitive access of the donor to the operating theatre to perform organ removal, and the case notification to the HTC (both dichotomous).

Independent variables were as follows:

- admission diagnosis (polytomous),
- cause of death (BD, asystole, or LLST),
- GCS score at admission lower or higher than 8 (dichotomous),
- hospital unit of admission or exitus (polytomous).

Intervening variables considered included the presence of absolute medical contraindications to donation, (family or judicial) authorization for the donation (both dichotomous), length of hospital stay until discharge or death, and socio-demographic age and sex variables. The age variable was categorized in years (any age range lower than 12 months old was indicated as 1 year), and the hospital stay length was categorized in days (stays lower than 24 h were taken as a day of admission); both were taken as interval variables. Independent polytomous variables were stratified according to the categorization included at the back of the data sheet about BD from the ONT PGCD [20].

### 2.6. Instrument

For the location of patients and their inclusion in the study, hospital records and applications belonging to the Clinic System of Documentation of CAULE were used:

- daily record of emergency admission,
- daily record of patients admitted to the hospital,
- daily record of deaths in the centre and in emergencies,
- Computerized System of Medical Histories (HIS) (Historia Clínica Digital del Sistema Nacional de Salud^®^, Salud Castilla y León, Spain),
- Computerized System of Nursing Care Management (Gacela Care^®^, Salud Castilla y León, Spain).

Indicators relating to the activity of the various CU were collected as were the PD generating units of CAULE, i.e., number of beds, number of deaths and emergencies attended by the hospital, and admissions in the CU and extra-CU units for the study period; the data were all provided by the CAULE Admission and Clinical Documentation Service.

The information needed for the study of the variables was collected in a questionnaire designed ad hoc, which included a specific item for each of the mentioned variables. These items were taken from the various forms used in the ONT PGCD. In addition, the age, sex, and length of hospital stay of each PD [15,17,18,20] were included.

The quality analysis of the process of detection of BD PD was conducted by comparing the results obtained with the standard criteria established by the ONT in their PGCD [15,17,18].

### 2.7. Data Collection Methodology

Available evidence was located in the Web of Science (WoS), Clinical Key, and Pubmed databases. Google Scholar hand search was done, and the CAULE HTC allowed the location of grey literature. The descriptors used were located in the DeCS and MeSH thesaurus: “organ donor”, “brain death”, “intensive care unit” and “emergency hospital service”. The terms “identification” and “coordination” were employed in their original language as they were not found in any thesaurus but were in fact indicated as search terms in the literature. Searches were conducted in Spanish and English.

To qualify a process such as CBD, there should be diagnostic accordance at the admission or death with at least one of the diagnostic codes listed in Table 1. The review of the medical history of each initially selected patient allowed the diagnosis of admission or death in concordance with these codes to be confirmed and therefore enabled its final inclusion in the sample as a case.

### 2.8. Statistical Analysis

For the statistical analysis, the free distribution software Epi Info (version 3.5.4, CDC, Atlanta, GA, USA) was employed. The contrast of hypotheses was performed by Student’s *t* and chi-square test for the univariate study and ANOVA test and nonparametric Kruskall–Wallis test for the bivariate analysis. A significant difference was shown when type I error probability was equal to or lower than 5%, which was assessed by confidence interval calculation (95% CI) and statistical Pearson *p*, with *p* ≤ 0.05 value for that probability.

### 2.9. Ethics Approval and Consent for Publication

The study was approved by the Ethics Committee in the Scientific Research Health Area of León, dated 20 December 2016 under record num. 16.117, with the CAULE Clinic Admission and Documentation Service written authorization for data collection of the patients included in the study and publication under anonymity.

## 3. Results

### 3.1. Activity of CAULE

During 2015, CAULE attended a population of 335,770 inhabitants. We took this fact as valid population imputation for the calculation of indicators as we did not have more up-to-date data at the conclusion of the fieldwork. During the study period, CAULE had an average of 827 beds; 44 of them had the ability to provide continuous ventilatory support to critical patients. The emergency services attended 64,611 patients of whom 17.4% (*n* = 11,264) (all specialties) were admitted and 81 died (0.12%). Table 2 shows the results relative to the number of beds, admissions, and exitus registered in each of the units studied and in the CAULE complex.

### 3.2. Studied Population

**Figure 1:**
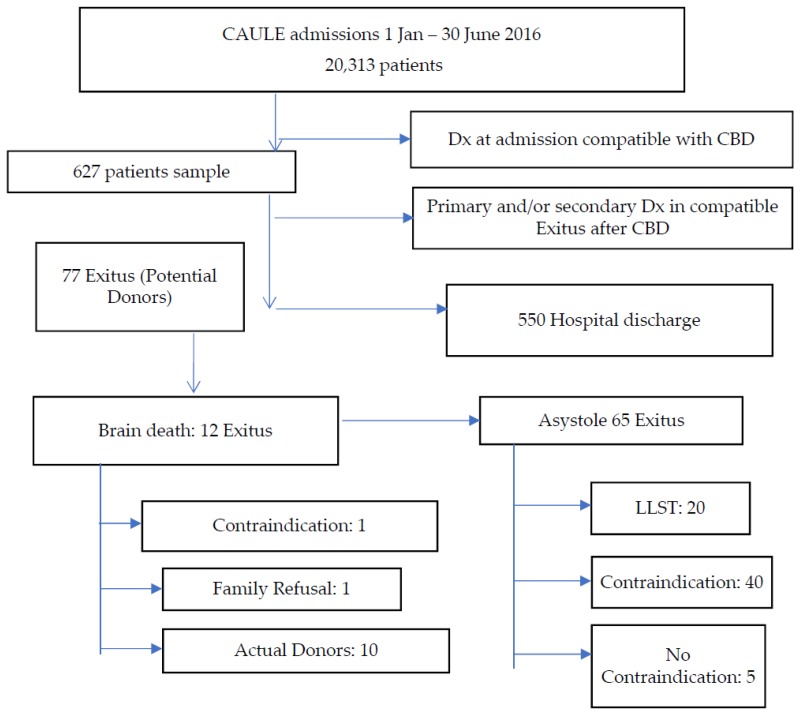
Sample inclusion criteria performance results.

The review of admissions and exitus that CAULE registered during the period of study allowed a total of 627 patients who met the first criterion for inclusion to be located; these constituted the reference population. After the application of the second criterion on this population, the final study sample was obtained, composed of 77 PD. The sample selection algorithm is shown in Figure 1 and can be summarized as follows: 87.7% of the patients (550/627) were discharged at home (*n* = 507), to another hospital (*n* = 13), or to a concerted centre (*n* = 30), and 12.3% (*n* = 77/627) of the initially selected patients died as a result of CBD.

The socio-demographic data are shown in Table 3. The most frequent sample age of the PD was 85 years old (*n* = 7), followed by PD aged 79 (*n* = 5), PD aged 83 (*n* = 5), and PD aged 90 (*n* = 5).

### 3.3. Analysis of the Quality Detection

The distribution of patients and PD according to the pathology of admission is presented in Table 4 and according to the admission unit in Table 5.

Of the PD sample, 84.4% (*n* = 65/77) died in asystole. Of these, 61 (79.2%) had absolute contraindications to donation. Five PD (EME = 4, COR = 1) had no contraindication, and they were notified to the HTC after hospital stays of a day (*n* = 4) and 90 days (*n* = 1). In all five of them, evidence of CBD was found. However, BD was not identified and could not be diagnosed. The option of organ donation was dismissed in the absence of the donation in asystole program, eventually dying of asystole after the LLST. Only two of these cases showed a GCS at admission below 8 (GCSi < 8).

Of the PD sample, 15.6% died of BD (*n* = 12/77), with 12.9% (*n* = 10/77) finally being effective donors. Among the 12 BD PD, a family refusal (8.3%) and an absolute contraindication to donation (8.3%) were recorded. The removal of organs took place on 10 occasions (83.3%), and there was no incidence of judicial refusal.

The bivariate analysis offered significant differences regarding the notification of cases of PD to the HTC according to the admission unit (*p* = 0.02). All (100%) of the RES (*n* = 2) and ICU (*n* = 21) registered PD was reported. Of those, 80% registered in extra-CU units (*n* = 12), leading to a reduction of those reported in EME to 65.5% (*n* = 19) and those in COR to 50% (*n* = 5).

No significant relationship (*p* = 0.07) was found between the presence of GCSi < 8 and a greater evolution probability of the PD detected as actual donors. This indicator of 69.9% of the PD was shown, with a record of the same (95% CI = 58.0% to 80.1%) (*n* = 51/73). Of these, actual donors were eventually 17.6% (*n* = 9/51), i.e., 90% (*n* = 9/10) of actual donors after BD showed a GCSi < 8.

Finally, indicators of the quality assessment of the detection process are shown in Table 6, only assessing those relating to this phase, within the ones included in the PGCD. The CAULE as a whole obtained indicators below the standard. However, in the ICU, they were above the standard. The quality of detection was above the stated standard.

## 4. Discussion

Several studies indicate that the cause of death in more than 95% BD organs donors is confined to a limited group of processes supported by the presence of CBD [10,15,16,28,29]. These processes are identified by the corresponding diagnostic code set stated in the International Classification of Diseases (ICD-10) of the World Health Organization [30]. This methodology offers 100% sensitivity in identifying potential donors based on the available evidence [29,31], and is the reason why it was selected for the detection of cases in this study. Its usefulness is obvious given the absence of BD without communication to the HTC.

As could be expected, the mortality rate in ICU is the largest of all the units studied. It is thus confirmed as the unit with the greatest potential for the generation of donors, as the available evidence shows [18,19,20,24,25,26,27]. However, it is necessary to take into account that some of the registered ICU exitus by CBD come from extra-CU units, such as NRL and especially EME. Therefore, whenever proceeding to obtain organs from donors in an authorized centre, the HTC must check the last will of the deceased on the National Registry of Prior Instructions (RNIP, for its acronym in Spanish). On the one hand, the HTC must investigate whether the donor, or his/her legal representative, communicated this will to relatives or professionals; on the other hand, the HTC must verify all the annotations made in the clinical history or in the relevant record regulated by law in Spain. This constitutes the expression of respect for the autonomy of people and allows the patient to decide on future healthcare decisions in their final stage of life, including the donation of organs [14]. Nevertheless, the PD detected in these units is usually assigned to ICU as an actual donor as appropriate.

This fact confirms the high potential for the identification of PD that extra-CU units possess, proven in various studies and operating recommendations [19,20,24,25]. Moreover, the same aforementioned evidence promotes HTC to keep a proactive attitude by daily reviewing and monitoring patients who are admitted or develop a CBD in these units, thus avoiding any loss of PD.

The high rate of detection in EME is attributable to the established synergies and to the agreed protocols between the HTC and this service following the latest recommendations [32]. Along the same lines, evidence suggests an agreement between training programs and periodic information to extra-CU unit professionals on the donation process and its results. These activities must focus on promoting a favorable attitude towards the donation issue among professionals and facilitating the incorporation of the option of donation to care provision at the end of a patient’s life [8,15,25].

On the other hand, the loss of five potential donors in asystole meant the loss of organs with the potential to be donated from cardiology critical patients who secondarily developed a CBD. In this sense, we agree with the evidence and point out the need to evaluate patients as BD PD [15,27]. The high probability of a CBD or asystole scenario in patients who have suffered a heart attack, or who suffer from a severe cardiac process, justifies the monitoring developed in this study to these patients as PD, both by BD and type II and III asystole [14,15,21,25,27,33,34].

Finally, the lack of statistical significance for the employment of GCSi < 8 value as a predictor of the greater chance that a detected PD will become an actual donor contrasts with what was found in the literature consulted. However, the available evidence suggests the use of this indicator in the detection of PD [15,24,25,32].

## 5. Conclusions

This study found that the detection process of potential organs donors conformed to the required quality standards from the PGCD and the National Transplant Organization.

However, population aging leads to a high rate of absolute contraindications among detected potential donors, affecting the final number of actual donors.

CAULE maintains its annual capacity of donation above the national target of 40 donors pmp, with an estimated capacity of almost 60 pmp per year. The profile type of donor is a 70-year-old male patient who dies of brain death after a severe stroke.

The loss of potential donors detected in asystole, mainly among the critical cardiac patients, has a decisive influence in the annual capacity of CAULE donation. Systematic follow-up of these patients as a potential source of donors, as well as the implementation of a donation program in controlled asystole cases that increases the donation capacity of this centre, is recommended.

## Figures and Tables

**Table 1:** ICE-10 Codes compatible with possible brain death cause.

General Causes	Specific Causes	
Head Injuries	S02	Fracture of skull and facial bones
	S061	Traumatic cerebral edema
	S062	Diffuse brain injury
	S063	Focal brain injury
	S064S	Epidural hemorrhage
	S067	Intracranial injury with prolonged coma
	S068	Other intracranial injuries
	S069	Intracranial injury, unspecified
Cerebrovascular	I60	Subarachnoid hemorrhage
	I61	Intracerebral hemorrhage
	I62	Other nontraumatic intracranial hemorrhage
	I63	Cerebral infarction
	I64	Stroke, not specified as hemorrhage or infarction
	I65	Occlusion and stenosis of precerebral arteries, not resulting in cerebral infarction
	I66	Occlusion and stenosis of cerebral arteries, not resulting in cerebral infarction
Other injuries	G931	Anoxic brain damage
	G935	Compression of brain
	G936	Cerebral edema
Brain Tumors	C71	Malignant neoplasm of brain
	D33	Benign neoplasm of brain and other parts of central nervous system
CNS infection	G00–G03	Meningitis

**Table 2:** Activity indicators of units generating potential donors *.

Study Unit	Beds	Admissions	Exitus	Mortality Rate
Coronary Unit	12	469	27	5.8%
Adults Intensive Care Unit	16	289	43	14.9%
Neurology Service/Stroke Unit	24	433	13	3.0%
Neurosurgery Service	28	527	7	1.3%
Postsurgery Resuscitation	12	592	24	4.1%
Pediatric Intensive Care Unit	4	193	0	0.0%
Internal Medicine Service/Others	192	2206	197	8.9%
Emergency Service	-	64,611	81	0.1%
CAULE TOTAL	827	20,313	727	3.6%

* Data corresponding to the study period: 1 January 2016–30 June 2016.

**Table 3:** Socio-demographic data.

Population Size	Reference Pop.	Potential Donors Samples
	*N* = 627	*N* = 77
Sample age (95% CI) *		
Average	68.0	75.9
Variance	314.1	291.5
Median ± Stand. Deviation	72.0 ± 17.6	80.0 ± 17.1
Minimum	59	15
Interquartile range (25–75%)	59a81	71a85
Maximum	96	96
Mode	79 (*n* = 29)	85 (*n* = 7)
Hospital Stay (95% CI) *		
Average	8.1	5.6
Variance	88.4	166.1
Median ± Standard Deviation	6.0 ± 9.4	2 ± 12.9
Minimum	1	1
Interquartile range (25–75%)	3a9	1a5
Maximum	124	90
Mode	4 (*n* = 75)	1 (*n* = 38)
Sex Distribution		
Males (95% IC)	61.9% (57.9a65.7)	54.5% (42.8a65.9)

* Age is expressed in whole years and hospital stay in days.

**Table 4:** Patient distribution according to admission pathology/exitus.

Admission Pathology	Reference Pop.		PD Sample	
	*n/N*	*%*	*n/N*	%
Acute Coronary Syndrome	223/627	35.6	17/77	22.1
Cerebrovascular Accident	298/627	47.5	38/77	49.4
Head injury	52/627	8.3	9/77	11.7
Other Brain Injuries (Anoxia, Edema)	17/627	2.7	9/77	11.7
Brain Tumors	17/627	2.7	1/77	1.3
Infection/Intoxication	20/627	3.2	3/77	3.9

PD = potential donors.

**Table 5:** Patient distribution according to case detection unit.

Case Location Unit	Reference Pop.		PD Sample	
	*n/N*	*%*	*n/N*	%
Coronary Unit	198/627	31.6	10/77	13.0
Adults Intensive Care Unit	66/627	10.5	21/77	27.3
Neurology Serv./Stroke Unit	222/627	35.4	8/77	10.4
Neurosurgery Serv.	66/627	10.5	4/77	5.2
Post-surgery Resuscitation	3/627	0.5	2/77	2.6
Paediatric Intensive Care Unit	16/627	2.5	0/77	0.0
Internal Medicine Serv./Others	27/627	4.3	3/77	3.8
Emergency Service	29/627	4.6	29/77	37.7

PD = potential donors.

**Table 6:** Quality indicators in the donation process *.

Capacity to Generate Donors	Quality	PD Sample	
(% According to Identified BD)	Standard	*n/N*	%
Exitus rate in Critical Unit (CU Exitus/CAULE Exitus)	>10%	84/727	11.5
CAULE BD incidence (BD/CAULE Exitus)	≈2.5%	12/727	1.7
BD incidence/CAULE N° of beds	>3.2%	12/827	1.5
Actual donors (AD)/CAULE 100 beds	>3.2%	10/827	1.2
AD/Critical U. 100 beds	>75%	10/44	22.7
AD/CAULE Exitus	>2.5%	10/727	1.4
AD/Critical U. Exitus	>10%	10/84	11.9
Donation rate (N° BD AD/CAULE area population) ×10^6^	≈40%	10/335.770	29.8
***Detection Process Optimization***			
Medical Contraindications/BD Exitus	<20%	1/12	8.3
BD with no detected contraindication (“losses”)/total BD	0.0%	0/12	0.0
Family refusal or no consent to donation	<10%	1/12	8.3
Judicial refusal to donation	<1%	0/12	0.0
Donation Success Rate (AD/BD PD)	>65%	10/12	83.3

* Data from the CAULE: 1 January 2016–30 June 2016. PD = potential donors. AD = actual donors. BD = brain death.

## Abbreviations

ONT	Organización Nacional De Trasplantes (National Transplant Organization)
PGCD	Programa De Garantía De Calidad En El Proceso De Donación (Quality Assurance Program In The Donation Process)
HTC	Hospital Transplant Coordinator
HIS	Computerized System of Medical Histories
BD	Brain Death
CAULE	Complejo Asistencial Universitario De León (University Assistance Complex of León)
CBD	Catastrophic Brain Damage
PD	Potential Donor
LLST	Limitation of Life Support Therapy
CCU	Critical Care Units
(P) ICU	(Pediatric) Intensive Care Unit
RES	Critical Postsurgical Resuscitation
COR	Critical Coronary Care Unit
NRL	Neurology Service Stroke Unit
NRS	Neurosurgery Service
IM	Internal Medicine Service
EME	Emergency Service
GCS	Glasgow Coma Scale
